# Characterization and Pathogenicity of Equine Herpesvirus Type 8 Using In-Vitro and In-Vivo Models

**DOI:** 10.3390/vetsci12040367

**Published:** 2025-04-15

**Authors:** Yanfei Ji, Dandan Xu, Wenxuan Si, Yu Zhang, Muhammad Zahoor Khan, Xia Zhao, Wenqiang Liu

**Affiliations:** College of Agriculture and Biology, Liaocheng University, Liaocheng 252000, China

**Keywords:** EHV-8, RK-13 cells cytopathic effects, pro-inflammatory cytokines, pathogenicity, BALB/c mice

## Abstract

Equine herpesvirus type 8 (EHV-8) is predominantly isolated from donkeys, but its biological properties and pathogenic potential remain underexplored. This study aimed to investigate the biological characteristics and pathogenicity of the EHV-8 LCDC01 isolate by examining its effects in rabbit kidney (RK-13) cells and BALB/c mice. The virus was assessed for its ability to induce viral replication, pathological changes, and alterations in pro-inflammatory responses. In vitro, the EHV-8 infection of RK-13 cells induced characteristic cytopathic effects, including cell contraction, the formation of grapevine bundle-like structures, and detachment. In vivo, mice infected with the virus exhibited no clinical signs other than weight loss. Polymerase chain reaction (PCR) analysis detected viral DNA exclusively in the lungs of infected mice, while TaqMan PCR further confirmed the presence of EHV-8 nucleic acids in the lungs, liver, brain, and intestines. Furthermore, ELISA assays revealed a significant increase in the secretion of pro-inflammatory cytokines, including IL-1β, IL-6, IL-8, and IFN-α, in the lungs (*p* < 0.05). These findings suggest that EHV-8 primarily replicates in the lung tissue of mice and can induce inflammatory responses. This study provides valuable insights into the pathogenic mechanisms of EHV-8 and lays the groundwork for further investigation into its potential impact on equine and other animal populations.

## Simple Summary

Equine herpesvirus 8 (EHV-8) is one of the most important pathogens affecting donkeys, but there are no studies on the transmission potential, pathogenicity, and immune response of systemic EHV-8. We investigated the pathogenicity and immune response to major target organs by studying EHV-8 infection in a BALB/c mouse model. The results showed that EHV-8 was able to effectively replicate and cause pathological changes in the lungs, liver, and brain and elicited an immune response in the organism, providing basic data for the study of the pathogenesis of EHV-8.

## 1. Introduction

Equine herpesviruses (EHVs) comprise nine distinct subtypes, taxonomically classified into the *α-herpesvirus* and *γ-herpesvirus* subfamilies. Within this classification, EHV-1, EHV-3, EHV-4, EHV-8, and EHV-9 are members of the *α-herpesvirus* subfamily, with EHV-1, EHV-4, and EHV-8 representing the most clinically significant viruses in equine populations [1,2,3]. EHV-8, which predominantly affects donkeys, has been implicated in multiple clinical manifestations, including respiratory disease, reproductive failure with abortion [3], and neurological disorders [4]. Notably, EHV-8 demonstrates serological cross-reactivity with both EHV-1 and EHV-4, complicating diagnostic differentiation.

Epidemiological surveillance has revealed that EHV-8 is endemic in large-scale donkey breeding operations across Shandong Province, China, exhibiting distinct seasonal epidemic patterns that impose a substantial economic burden on the expanding donkey farming industry [5]. The rapid growth of donkey husbandry in China has elevated the importance of understanding EHV-8 pathobiology, rendering its study particularly relevant in comparison to EHV-1 and EHV-4 within this regional context.

Despite this increasing significance, research on EHV-8 remains comparatively limited, with the existing literature primarily focused on virus isolation, identification, and genomic characterization. Consequently, fundamental aspects of EHV-8 pathogenesis remain poorly elucidated, representing a critical knowledge gap. Previous investigations have utilized rabbit kidney epithelial (RK-13) cell culture systems followed by murine models to evaluate potential therapeutic interventions against EHV-8 infection [6]. Building upon this foundation, the present study examines the pathogenicity and inflammatory responses induced by EHV-8 in both RK-13 cells and a mouse model. The findings reported herein provide essential insights that may serve as a foundation for subsequent investigations into the molecular mechanisms governing EHV-8 infection and host-pathogen interactions.

## 2. Materials and Methods

### 2.1. Virus, Cells, and Mice

The EHV-8 strain LCDC01 (GenBank accession: PRJNA787358) was isolated in 2021 from nasal swabs collected from infected donkeys at a large-scale donkey farm in Liaocheng, China. Virus isolation was accomplished through the inoculation of RK-13 cells. The RK-13 cell lines were obtained from Wuhan Punosai Life Science and Technology Co., Ltd. (Wuhan, China). Cells were maintained in Dulbecco’s Modified Eagle Medium (DMEM) supplemented with 10% fetal bovine serum (FBS). All cell culture reagents, including DMEM, FBS, and cell cryopreservation solution, were sourced from Dalian Meilun Biotechnology Co., Ltd. (Dalian, China). Specific pathogen-free (SPF) female BALB/c mice aged 2–3 weeks were used for the in vivo experiments. All mice were obtained from Jiangsu Huachuang Xinuo Pharmaceutical Technology Co., Ltd. (Taizhou, Jiangsu, China).

### 2.2. Viral Inoculation of Cells

RK-13 cells were cultured in 25 cm^2^ flasks until approximately 80% confluence was reached. The virus was inoculated by adding 200 μL of EHV-8 virus suspension to the cells, followed by 800 μL of MEM medium (Meilun, Dalian, China). After a 1-h incubation at 37 °C, the viral inoculum was removed, and fresh complete medium (5 mL) was added. The cells were incubated overnight and periodically observed for cytopathic effects (CPE). Once CPE was apparent, the supernatant was collected to observe the viral morphology using a transmission electron microscope (H-7650, Hitachi Ltd., Tokyo, Japan).

### 2.3. Determination of TCID_50_

RK-13 cells (1 × 10^7^ Cells/mL) were seeded in 96-well plates and cultured at 37 °C in a 5% CO_2_ atmosphere for 24 h. After reaching approximately 80% confluence, the culture medium was discarded. Virus inoculum derived from the original strain propagated in RK-13 cells (as described in Section 2.2) was subjected to serial 10-fold dilutions in MEM. Each dilution (100 μL) was added to wells in rows 1–11, while row 12 served as a negative control containing only MEM. Following incubation at 37 °C in a 5% CO_2_ atmosphere for 1 h, the inoculum was removed and replaced with fresh complete medium. Cells were monitored daily for cytopathic effects (CPE). After 48 h post-infection (hpi), TCID_50_ values were calculated based on the number of wells exhibiting CPE using the Reed–Muench method [7].

### 2.4. Immunofluorescence Detection of EHV-8 Antibody

An indirect immunofluorescence assay was performed according to the protocol described by Broeck et al. [8]. Following cell fixation, donkey anti-EHV-8 positive serum (diluted 1:100 in PBS) was applied (200 μL per well) and incubated for 2 h at room temperature. After three washes with PBS, FITC-conjugated rabbit anti-donkey secondary antibody (ImmunoWay, Plano, TX, USA) was added at a 1:1000 dilution and incubated for 1 h in darkness. Cells were subsequently mounted with 50 μL of 50% glycerol in blocking solution. The observation of fluorescence was made using a fluorescence microscope (Nikon Eclipse C1, Tokyo, Japan).

### 2.5. Apoptosis Detection

Apoptosis in EHV-8-infected RK-13 cells was assessed when approximately 30% of cells exhibited CPE using an Annexin V-FITC/ propidium iodide (PI) Apoptosis Detection Kit (Meron Biotechnology, Tianjin, China) according to the manufacturer’s protocol. Briefly, adherent cells were harvested using EDTA-free trypsin, centrifuged at 1000× *g* for 5 min, and washed twice with cold PBS. Cell pellets were resuspended in 500 μL binding buffer, and 5 μL each of Annexin V-FITC and PI were added. Following gentle mixing and incubation for 15 min at room temperature in darkness, samples were analyzed by fluorescence microscopy.

### 2.6. Virus Infection and Sampling of Mice

BALB/c mice were randomly divided into two experimental groups: virus-infected (n = 15) and control (n = 15). Prior to inoculation, mice were anesthetized with CO_2_ for 20 s. The virus-infected group received 50 μL of EHV-8 viral suspension (TCID_50_ = 10^−3.75^/100 μL) intranasally, while the control group was administered 50 μL of sterile saline via the same route. Animals were monitored daily for clinical manifestations, and body weight was recorded at regular intervals. Blood samples were collected via retro-orbital puncture under appropriate anesthesia at 12 h, 1, 3, 5, and 7 days post-infection to evaluate the progression of EHV-8 infection. The serum was isolated by centrifugation of whole blood at 3000 × g for 10 min at 4 °C. At each time point, three mice from each group were euthanized by cervical dislocation. Pulmonary tissues were harvested for determination of the lung index, defined as the ratio of lung weight to body weight [9].

Lung index = (Lung weight (g) Body weight (g)) × 100 Lung index = (Body weight (g) Lung weight (g)) × 100

At 5 dpi, heart, liver, spleen, kidney, brain, and intestinal tissues were also collected for viral replication analysis to determine the damage of 5 days post-infection (dpi) to tissues and organs in mice. The lungs, liver, and brain were fixed with 4% formaldehyde and stained under the microscope according to the instructions of the HE (Hematoxylin-Eosin) Stain Kit (Solarbio, Beijing, China).

### 2.7. Determination of Viral Load in Major Organs of Mice

To determine the viral load at 5 dpi, we collected tissue samples from the heart, liver, spleen, lungs, kidneys, brain, and intestines from infected mice. Viral DNA (30 μL) was extracted from these 50 mg tissue samples using the AxyPrep Somatic Fluid Virus DNA/RNA Miniprep Kit (Corning Incorporated, New York, NY, USA). The extracted DNA was then analyzed by conventional PCR and quantitative PCR [10]. PCR primers for both conventional and quantitative PCR were designed based on the EHV-8 gB gene (GenBank accession: DVGE214585JN), with primer synthesis by Shanghai Bioengineering Co., Ltd. (Shanghai, China). The sequences of the primers are listed in Table 1.

The PCR reaction was performed in a total volume of 25 μL containing 12.5 μL of M5 Hiper Plus Taq HiFi DNA polymerase master mix (Mei5bio, Beijing, China), 1 μL of each primer (EHV-8-F and EHV-8-R, 10 μM each), 8.5 μL of nuclease-free water, and 2 μL of template DNA. Thermal cycling conditions were as follows: initial denaturation at 95 °C for 5 min; followed by 30 cycles of denaturation at 95 °C for 30 s, annealing at 60 °C for 30 s, then extend at 72 °C for 1 min; finally extend at 72 °C for 10 min.

For qPCR analysis, reactions were carried out in a 25 μL volume containing 12.5 μL of Premix Ex Taq (Takara, Japan), 1 μL of each primer (EHV-8-F and EHV-8-R, 10 μM each), 1 μL of EHV-8 probe (EHV-8-P, 10 μM), 7.5 μL of nuclease-free water, and 2 μL of template DNA. The qPCR thermal cycling parameters were: initial denaturation at 95 °C for 50 s, followed by 40 cycles of denaturation at 95 °C for 5 s and annealing/extension at 60 °C for 30 s. Cycle threshold (Ct) values were determined at the completion of the reaction and calculated based on the standard curve. Viral load quantification was achieved using a standard curve generated from serial dilutions of known concentrations of EHV-8 DNA.

### 2.8. Serological Detection of EHV-8 Antibodies

According to the EHV-8 gD antibody assay developed in our laboratory [11], mouse serum was applied to ELISA plates coated with polyclonal antibody to EHV-8 gD protein. The assay was performed in triplicate, and absorbance values were recorded at 450 nm.

### 2.9. Cytokine Detection

Mouse serum samples were analyzed for IL-1β, IL-6, IL-8, and IFN-α cytokine levels using Jang Su Enzyme-Linked Immunoassay kits and Enzyme-Linked Immunosorbent Assay (ELISA) kits following the manufacturer’s protocols (Meimian, Yancheng, China). All assays were performed in technical triplicate. Absorbance values were measured at 450 nm (OD450) using a microplate reader to quantify cytokine concentrations.

### 2.10. Data and Analytics

Experimental data were counted and analyzed using SPSS statistics 23 (IBM, New York, NY, USA) and graphically analyzed using GraphPad prism 8.0.2 (GraphPad Software, Boston, MA, USA). Data are expressed as mean ± standard deviation (n = 3). Differences between the two groups were analyzed using the *t*-test, with “ns” indicating a non-significant difference *p* > 0.05, “*” indicating a significant difference *p* < 0.05, and “**” indicating a significant difference *p* < 0.01.

## 3. Results

### 3.1. Identification of the Virus

**Figure 1:**
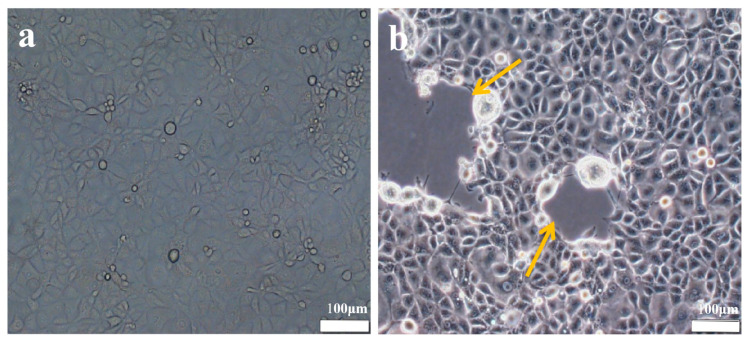
Cytopathological changes of EHV-8 infection (40×). (**a**): Control group, normal growth pattern of RK-13 cells in normal group. (**b**): EHV-8 infection group, orange arrow: EHV-8 infection of RK-13 cells with CPE phenomenon.

**Figure 2:**
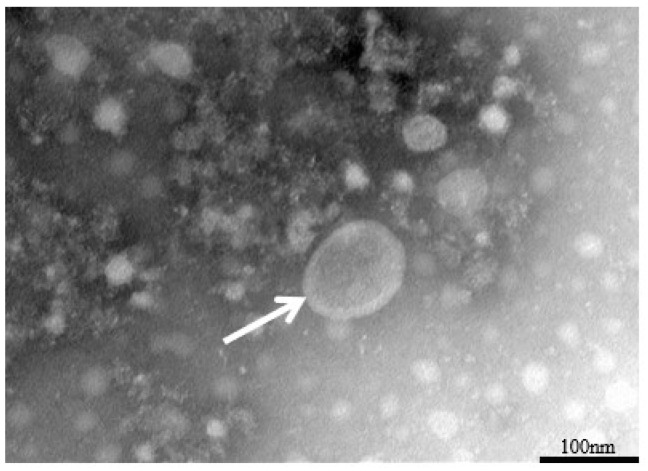
Electron microscopic observation of virus (×80.0 k). White arrow: EHV-8 virus particles.

The EHV-8 strain was inoculated into monolayers of RK-13 cells and monitored daily via microscopy. After 48 h, the control group is normal (Figure 1a), and the infection group shows typical cytopathic effects, including cell rounding, fusion, and detachment (Figure 1b, orange arrow). Additionally, as shown in Figure 2, viral particles were examined using transmission electron microscopy, revealing particles approximately 100 nm in diameter, with a nearly circular shape and an outer vesicular envelope.

### 3.2. Viral Titer Measurement

The viral stock was diluted at 1:10 and inoculated into RK-13 cells cultured in a 96-well plate. The CPE was monitored continuously, and wells exhibiting more than 50% CPE were counted. After 48 hpi, the determined TCID_50_ of the isolated strains was TCID_50_ = 10^−3.75^/100 μL.

### 3.3. Immunofluorescence Assay

**Figure 3:**
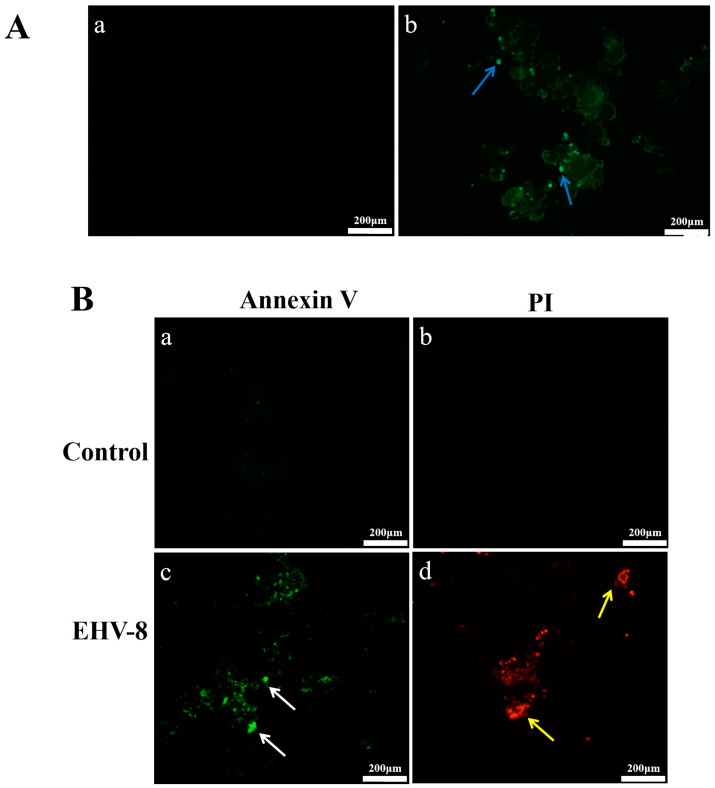
EHV-8 antibody test results and Apoptosis of EHV-8 infected cells and summary of immune responses (40×). (**Aa**) Indirect immunization with control EHV-8 antibody. (**Ab**) Indirect immunization with control EHV-8 antibody, blue arrows: EHV-8 binds to antibodies. (**Ba**) Control group: Annexin V fluorescence analysis of RK-13 cells. (**Bb**) Control group: Propidium iodide (PI) fluorescence analysis of RK-13 cells. (**Bc**) EHV-8 infection group: Annexin V fluorescence analysis of RK-13 cells, White arrows: early apoptotic cell fluorescence. (**Bd**) EHV-8 infection group: Propidium iodide (PI) fluorescence analysis of RK-13 cells yellow arrows: fluorescence of necrotic or advanced apoptotic cells.

Figure 3 demonstrates EHV-8 infection and its effects on RK-13 cells. In Figure 3A, immunofluorescence analysis revealed abundant EHV-8-specific fluorescence in infected cells (Figure 3(Ab)), while control cells exhibited no detectable signal (Figure 3(Aa)), confirming efficient viral infection and replication in RK-13 cells. Figure 3B illustrates apoptotic events following viral infection. Early apoptosis, visualized by green fluorescence (white arrows), was markedly increased in infected cells (Figure 3(Bc)) compared to uninfected controls (Figure 3(Ba)). Similarly, late-stage apoptosis or necrosis, indicated by red fluorescence (yellow arrows), was substantially higher in infected cells (Figure 3(Bd)) than in control cells (Figure 3(Bb)). These results demonstrate that EHV-8 infection effectively induces apoptosis in RK-13 cells. 

### 3.4. Clinical and Pathological Changes in Mice

#### 3.4.1. Effect of EHV-8 Infection on Body Weight in Mice

**Figure 4:**
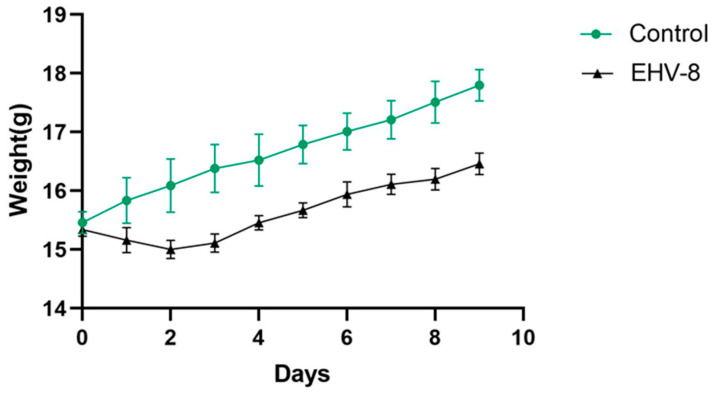
The line graph of body weight changes in mice. Note: Data are expressed as mean ± SEM (Standard Error of the Mean), and a *t*-test was used for differences between the two groups.

Following intranasal infection, no significant clinical symptoms were observed in the mice except for weight loss. Mice in the virus-infected group exhibited a gradual decrease in body weight from days 1 to 3 post-inoculation (dpi). From day 4 onwards, growth resumed, although at a slower rate compared to the control group. At 5 dpi, the growth rate of the infected mice returned to normal, while the control group continued to show normal weight gain (Figure 4).

#### 3.4.2. Effect of EHV-8 Infection on Lung Indices in Mice

**Figure 5:**
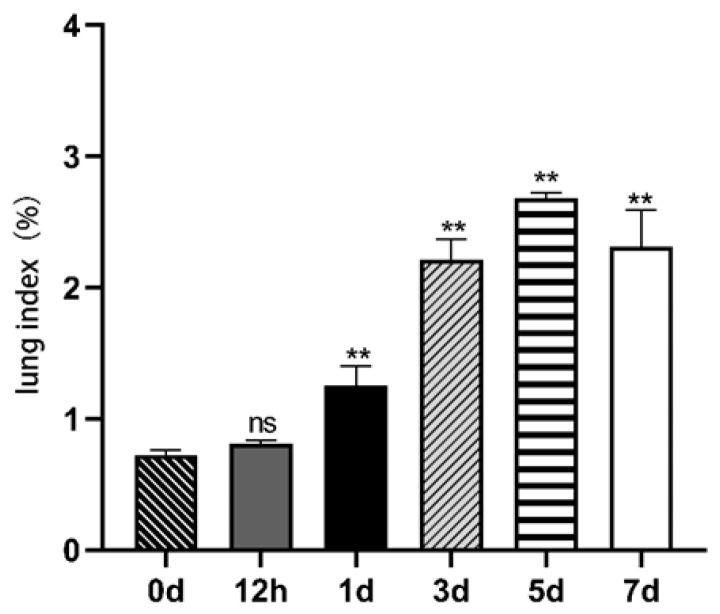
Lung indices at different times in EHV-8 infected mice. Note: Data are expressed as mean ± SEM (Standard Error of the Mean), and *t*-test was used for differences between the two groups, ‘**’ indicates a significant extreme difference compared to the control group (*p* < 0.01); ‘ns’ indicates an insignificant difference compared to the control group (*p* > 0.05). (0 d): Lung index in control mice. (12 h): 12 h Lung index of mice infected for 12 h in EHV-8-infected group. (1 d): Lung index in mice infected with EHV-8 in the EHV-8-infected group for 1 day of infection. (3 d): Lung index of mice infected for 3 days in the EHV-8-infected group. (5 d): Lung index in mice infected with EHV-8 in the EHV-8-infected group for 5 days of infection. (7 d): Lung index of mice infected for 7 days in the EHV-8-infected group.

As shown in Figure 5, there were no significant changes in the lung index at 12 h post- EHV-8 infection (*p* > 0.05). However, by day 5 post-infection, the lung index reached its peak at 2.48%, showing a highly significant difference compared to the control group (*p* < 0.01).

#### 3.4.3. Pathological Observations of EHV-8 Infection on Mouse Organs

**Figure 6:**
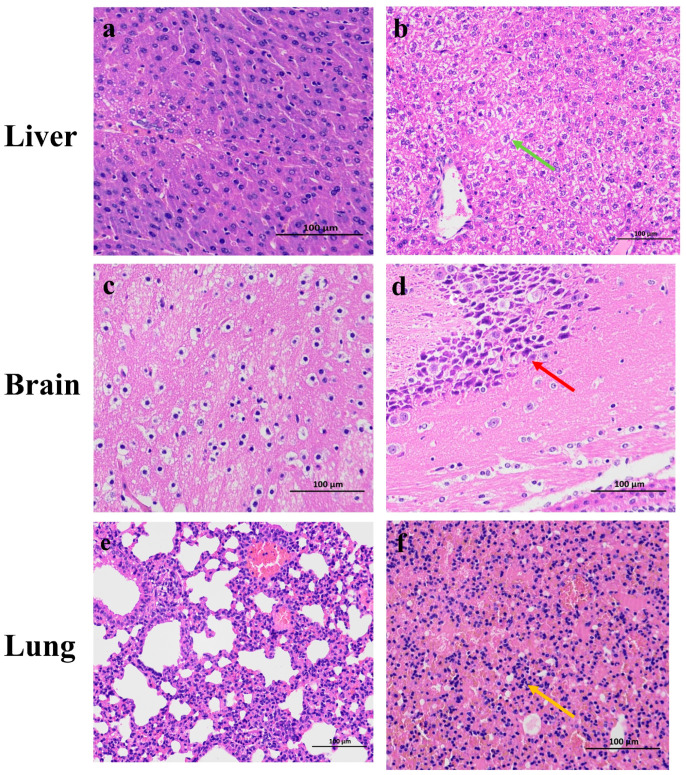
HE staining of various organs in the EHV-8 infected mice (40×). (**a**) HE staining of liver from mice in the control group. (**b**) HE staining of liver from mice in the EHV-8 infection group, green arrow: intravascular congestion, mild steatosis around the portal area, occasional vesicular vacuoles in the cytoplasm, and small clumps of bluish-purple granular material. (**c**) HE staining of brain from mice in the control group. (**d**) HE staining of brain from mice in the EHV-8 infection group, red arrow: vacuolization in the cytoplasm of nerve cells. (**e**) HE staining of lung from mice in the control group. (**f**) HE staining of lung from mice in the EHV-8 infection group, orange arrow: extensive thickening of the alveolar walls with numerous infiltrating inflammatory cells.

Pathological examination at 5 dpi revealed significant histopathological alterations in multiple organs of infected mice compared to controls. In the liver, control animals exhibited normal hepatic architecture (Figure 6b, green arrows), whereas infected mice demonstrated marked intravascular congestion, mild periportal steatosis, occasional cytoplasmic vacuolation in venous structures, and focal deposits of basophilic granular material. Notably, inflammatory cell infiltration was minimal in hepatic tissues. In the brain, control animals displayed normal neuronal morphology and organization (Figure 6d, red arrows). In contrast, infected mice exhibited numerous hippocampal neurons with hyperchromatic nuclei and disrupted cellular arrangement. Cytoplasmic vacuolization was prominent in neuronal cells, though glial proliferation was not observed. Pulmonary tissues from control animals maintained normal alveolar architecture, while infected mice demonstrated extensive alveolar septal thickening with significant inflammatory cell infiltration (Figure 6f, orange arrows).

#### 3.4.4. Distribution of EHV-8 Infected Mice in Different Organs and at Different Times of the Day

**Figure 7:**
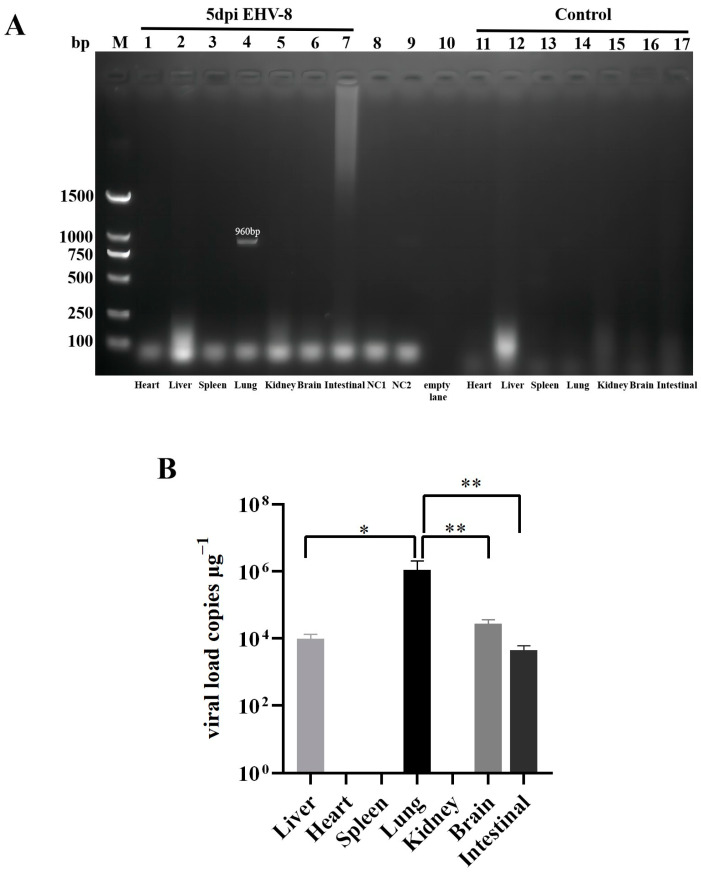
Levels of EHV-8 in mouse tissues after 5 d of infection. Note: Data are expressed as mean ± SEM (Standard Error of the Mean), and t-test was used for differences between the two groups, ‘*’ indicates a significant difference compared to the control group (*p* < 0.05); ‘**’ indicates a significant extreme difference compared to the control group (*p* < 0.01). (**A**): PCR result, samples are representative of each group, M: 2000 DNA maker; NC1: Negative control 1, NC2: Negative control 2. (**B**): 48 h EHV-8 group quantitative PCR result.

As demonstrated in Figure 7A, a 960 bp target band was detected exclusively in the lungs of mice at 5 dpi. No target bands were observed in other organs or control samples, confirming successful detection of EHV-8 nucleic acid specifically in the lungs of infected mice. Figure 7B illustrates the quantitative detection of EHV-8 viral load using fluorescence quantitative PCR. Viral DNA was detected in the liver, lung, brain, and intestine of 5 dpi mice, while remaining undetectable in all control samples. Notably, viral load in the lungs was significantly higher compared to the liver, brain, and intestine, suggesting preferential viral replication in pulmonary tissue.

### 3.5. Temporal Dynamics of Anti-EHV-8 Immune Response

**Figure 8:**
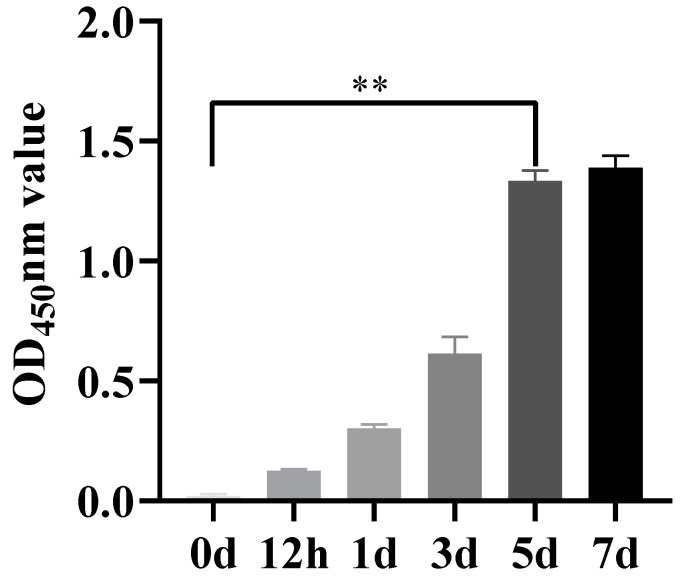
EHV-8 antibody level in mouse serum. Note: Data are expressed as mean ± SEM (Standard Error of the Mean), and t-test was used for differences between the two groups, ‘**’ indicates a significant extreme difference compared to the control group (*p* < 0.01); (0 d): Control group. (12 h): Samples from 12 h of EHV-8 infection. (1 d): Samples from 1 day of EHV-8 infection. (3 d): Samples from 3 days of EHV-8 infection. (5 d): Samples from 5 days of EHV-8 infection. (7 d): Samples from 7 days of EHV-8 infection.

As shown in Figure 8, according to the EHV-8 gD antibody detection method, OD_450_ nm greater than 0.269 was judged as positive, after the mice were infected with EHV-8, the concentration of EHV-8 antibody increased gradually with the growth of time, and the concentration of antibody increased rapidly at 3–5 dpi, and there was a significant difference between the EHV-8 antibody concentration control group at 5 dpi, which indicated that EHV-8 was in the body of the mice, replicated, and induced an immune response in mice.

### 3.6. Cytokine Expression Patterns Following EHV-8 Infection

**Figure 9:**
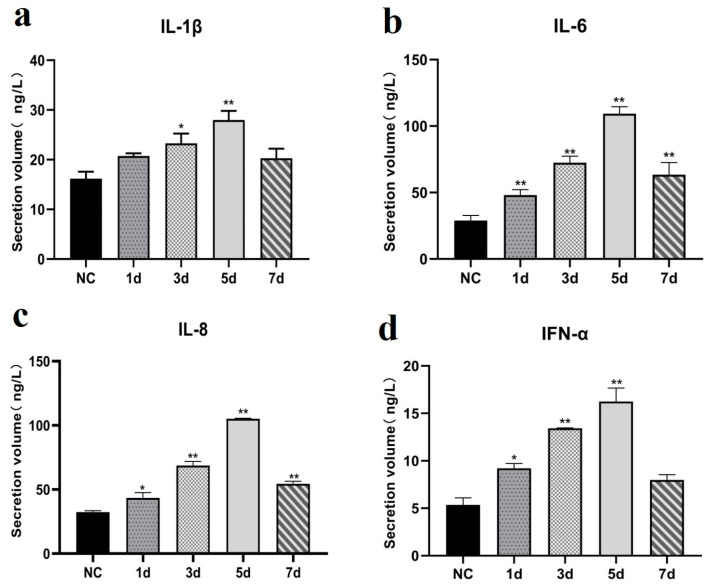
ELISA plot of immune factors for different days of EHV-8 infection. Note: Data are expressed as mean ± SEM (Standard Error of the Mean), and *t*-test was used for differences between the two groups, ‘*’ indicates a significant difference compared to the control group (*p* < 0.05); ‘**’ indicates a significant extreme difference compared to the control group (*p* < 0.01); (**a**): IL-1β ELISA result. (**b**): IL-6 ELISA result. (**c**): IL-8 ELISA result. (**d**): IFN-α ELISA result. (NC): negative control group. (1 d): Samples from 1 day of EHV-8 infection. (3 d): Samples from 3 days of EHV-8 infection. (5 d): Samples from 5 days of EHV-8 infection. (7 d): Samples from 7 days of EHV-8 infection.

As shown in Figure 9, the secretion of cytokines, including IL-1β, IL-6, IL-8, and IFN-α, was significantly elevated in the lungs of infected mice compared to the control group (*p* < 0.05). The cytokine levels increased markedly at 3 dpi, peaked at 5 dpi, and subsequently decreased by 7 dpi.

## 4. Discussion

In the present investigation, we sought to comprehensively characterize viral proliferation, histopathological manifestations, and inflammatory responses in both RK-13 cells and a murine model following infection with the LCDC01 isolate. Upon infection, the LCDC01 strain consistently induced characteristic CPE and apoptotic changes in RK-13 cells, thereby providing compelling evidence of active viral replication within these cellular substrates. Notably, despite the absence of overt clinical manifestations in the infected mice, a significant reduction in body weight was documented, which strongly suggests that the LCDC01 strain possesses modest toxicity and demonstrates relatively low virulence in vivo.

The detailed histopathological examination of various tissue sections revealed differential patterns of inflammatory damage across organs, with pulmonary tissues exhibiting particularly pronounced pathological alterations compared to hepatic and cerebral tissues. These observations are in concordance with previously published findings regarding EHV-1-induced tissue pathology in murine models [12,13]. Furthermore, molecular analysis using PCR for the detection of EHV-8 nucleic acid yielded positive results exclusively in pulmonary specimens, whereas all other examined organs generated negative findings. This apparent discrepancy strongly indicates that the virus preferentially replicates within pulmonary tissues, with substantially reduced viral burdens in extrapulmonary sites. It is important to note that the inherent sensitivity limitations of conventional PCR methodology may preclude the detection of viral genomic material in tissues harboring low viral loads, a hypothesis supported by the positive results subsequently obtained using more sensitive quantitative PCR assays.

Following the experimental inoculation of RK-13 cells with the LCDC01 isolate, we observed the development of characteristic CPE at 48 hpi, with a calculated TCID_50_ of 10^−3.75^ mL^−1^. These values are consistent with previously reported metrics for EHV-1-induced cytopathology in comparable experimental systems [14]. Ultrastructural analysis using transmission electron microscopy (TEM) revealed the presence of typical herpesvirus virions measuring approximately 100 nm in diameter, which aligns precisely with the established morphological characteristics of EHV-like viruses described in the literature [15].

The complex interplay between viral infection and host immune response is critical in determining disease progression and severity. Thus, cytokines such as IL-1β, IL-6, IL-8, and IFN-α play fundamental roles in orchestrating the immune system’s multifaceted response to injury, infection, and malignancy. Each of these signaling molecules contributes distinctively to immune regulation, tissue repair, and pathogenesis across various physiological contexts [16,17]. Consistent with previous research demonstrating that EHV-1 infection triggers upregulation of pro-inflammatory cytokines in murine models [18], our investigation revealed significant elevations in IL-1β, IL-6, IL-8, and IFN-α levels in both the serum and pulmonary tissues of mice infected with the LCDC01 strain. Importantly, the secretion of these inflammatory mediators showed marked increases, specifically on days 3 and 5 post-infection. This temporal pattern strongly suggests that the LCDC01 strain not only induces lung injury but also elicits a robust inflammatory cascade in the pulmonary tissues of infected mice, which is in line with previously reported research [6]. Collectively, these findings provide valuable insights into the pathogenic mechanisms underlying EHV-8 infection and highlight the pivotal role of inflammatory cytokines in viral pathogenesis. The temporal correlation between peak cytokine levels and histopathological damage underscores the potential contribution of immune-mediated mechanisms to tissue injury. Further investigations are warranted to elucidate the precise molecular pathways involved in EHV-8-mediated tissue damage and immune dysregulation, which may ultimately inform therapeutic strategies targeting specific components of the inflammatory response.

## 5. Conclusions

The EHV-8 represents a significant pathogen affecting donkeys with considerable economic implications for the donkey industry. In this study, we found that the LCDC01 strain EHV-8 was able to cause cytopathic effects and apoptosis in RK-13 cells. Upon infection in the murine model, EHV-8 caused substantial pathological damage to hepatic, neural, and pulmonary tissues, with concurrent elevation of serum immune mediators. Notably, pulmonary tissue exhibited the highest viral load among examined organs. These findings provide a foundational framework for understanding EHV-8 pathogenesis and host-pathogen interactions, which may inform future preventive and therapeutic strategies against this economically important equid herpesvirus.

## Figures and Tables

**Table 1:** Primer Sequence Information.

Name	Sequences
PCR-Forward primer (F)	5′-TGTGAAAAATTCA AACGT-3′
PCR-Reverse primer (R)	5′-GAAGGTGCTGTTGCTTTTGCTGG-3′
Quantitative PCR-F	5′-GCGAACCCTCTGGAACGAAA-3′
Quantitative PCR-R	5′-TGGCTATCACGTCTCCCAGG-3′
Quantitative PCR-P	5′-ACTCTTGACGAGCGAGTTGCGGCGA-3′

## Data Availability

The data supporting the conclusions of this case report are included in this article. All data sets can be requested from correspondence with the authors.

## References

[1] El Brini Z., Cullinane A., Garvey M., Fassi Fihri O., Fellahi S., Amraoui F., Loutfi C., Sebbar G., Paillot R., Piro M. (2025). First Molecular and Phylogenetic Characterization of Equine Herpesvirus-1 (EHV-1) and Equine Herpesvirus-4 (EHV-4) in Morocco. Animals.

[2] Worku A., Molla W., Kenubih A., Negussie H., Admassu B., Ejo M., Dagnaw G.G., Bitew A.B., Fentahun T., Getnet K. (2024). Molecular Detection of Equine Herpesviruses from Field Outbreaks in Donkeys in Northwest Amhara Region, Ethiopia. Vet. Med. Int..

[3] Ruan L., Li L., Yang R., You A., Khan M.Z., Yu Y., Chen L., Li Y., Liu G., Wang C. (2025). Equine Herpesvirus-1 Induced Respiratory Disease in Dezhou Donkey Foals: Case Study from China, 2024. Vet. Sci..

[4] Schvartz G., Edery N., Moss L., Hadad R., Steinman A., Karniely S. (2020). Equid Herpesvirus 8 Isolated from an Adult Donkey in Israel. J. Equine Vet. Sci..

[5] Chen L., Li S., Li W., Yu Y., Sun Q., Chen W., Zhou H., Wang C., Li L., Xu M. (2024). Rutin prevents EqHV-8 induced infection and oxidative stress via Nrf2/HO-1 signaling pathway. Front. Cell. Infect. Microbiol..

[6] Hu L., Wang T., Ren H., Liu W., Li Y., Wang C., Li L. (2022). Characterizing the Pathogenesis and Immune Response of Equine Herpesvirus 8 Infection in Lung of Mice. Animals.

[7] Mizuno M., Kimbara S., Ichise H., Ishikawa N., Nishihara Y., Nishio M., Sekiya I. (2025). Cleaning methods for biosafety cabinet to eliminate residual mycoplasmas, viruses, and endotoxins after changeover. Regen. Ther..

[8] Broeckl C.V., Hiereth S., Straubinger R.K. (2024). A comparative study evaluating three line immunoassays available for serodiagnosis of equine Lyme borreliosis: Detection of Borrelia burgdorferi sensu lato-specific antibodies in serum samples of vaccinated and non-vaccinated horses. PLoS ONE.

[9] Wu X., Xu L., Xu G., Xu Y., Liu H., Hu Y., Ye X., Huang Q., Tang C., Duan N. (2025). Fei-yan-qing-hua decoction exerts an anti-inflammatory role during influenza by inhibiting the infiltration of macrophages and neutrophils through NF-κB and p38 MAPK pathways. J. Ethnopharmacol..

[10] Ji Y., Qi L., Zhang J., Xu D., Zhang J., Zhao X., Liu W. (2023). Establishment of TaqMan Real-time Fluorescence Quantitative PCR Detection Method for Equine Herpesvirus Type 8. Chin. Vet. J..

[11] Zhang J. (2022). Epidemiological Survey of Equine Herpesvirus in Large-Scale Donkey Farms and Preparation of gD Protein Polyantibodies of EHV-8 Isolates.

[12] Kimura H., Shibata M., Kuzushima K., Nishikawa K., Nishiyama Y., Morishima T. (1990). Detection and direct typing of herpes simplex virus by polymerase chain reaction. Med. Microbiol. Immunol..

[13] Walker C., Love D.N., Whalley J.M. (1999). Comparison of the pathogenesis of acute equine herpesvirus 1 (EHV-1) infection in the horse and the mouse model: A review. Vet. Microbiol..

[14] Gosztonyi G., Borchers K., Ludwig H. (2010). Pathogenesis of equine herpesvirus-1 infection in the mouse model. Apmis.

[15] Tong P., Pan J., Dang Y., Yang E., Jia C., Duan R., Tian S., Palidan N., Kuang L., Wang C. (2024). First identification and isolation of equine herpesvirus type 1 in aborted fetal lung tissues of donkeys. Virol. J..

[16] Heinrich P.C., Behrmann I., Haan S., Hermanns H.M., Müller-Newen G., Schaper F. (2003). Principles of interleukin (IL)-6-type cytokine signalling and its regulation. Biochem. J..

[17] Krumm B., Xiang Y., Deng J. (2014). Structural biology of the IL-1 superfamily: Key cytokines in the regulation of immune and inflammatory responses. Protein Sci..

[18] Shi W., Yao X., Fu Y., Wang Y. (2022). Interferon-α and its effects on cancer cell apoptosis (Review). Oncol. Lett..

